# Social Media as a Driver of Obesity in Children and Adolescents (Aged 6-18 Years): It Is Time for Regulatory Action

**DOI:** 10.2196/95678

**Published:** 2026-08-10

**Authors:** Yuwei Liu, Wenyun Li, Li Ming Wen, Gengsheng He

**Affiliations:** 1 School of Public Health, National Health Commission Key Laboratory of Health Technology Assessment, Fudan University, 130 Dong’an Road, Shanghai, 200032, China, 86 2154237271; 2 Department of Clinical Nutrition, Zhongshan Hospital, Fudan University, Shanghai, China; 3 School of Public Health, Faculty of Medicine and Health, The University of Sydney, NSW, Australia

**Keywords:** social media, childhood obesity, digital food marketing, online platform governance, artificial intelligence

## Abstract

Obesity in children and adolescents is rising in China and globally, with health consequences that are already evident during childhood. This Viewpoint represents the authors’ interpretation of current evidence and policy experience, using China as an illustrative case for a wider international challenge. We argue that social media should be considered a modifiable obesogenic environment because it can amplify sedentary behavior, digital food marketing, disrupted sleep, body image pressures, cyberbullying-related distress, and reward-based design that encourages prolonged device use. The aim of this Viewpoint is to explain why proportionate regulation of social media should be included in childhood obesity prevention. We make 4 key arguments: digital food marketing and persuasive platform design require stronger age-sensitive governance; regulatory approaches should be differentiated by developmental stage and social context; AI-assisted moderation may support enforcement but requires safeguards; and regulation should complement, not replace, school-based education, family engagement, and healthy technology design. The intended audience includes public health researchers, clinicians, policymakers, platform regulators, educators, and child-health advocates in China and internationally.

## Introduction

Childhood obesity has emerged as a global health concern with substantial short- and long-term health and societal consequences [1]. In this article, childhood obesity refers to obesity among children and adolescents aged 6 to 18 years. We focus on this age range because it corresponds to school-aged children and adolescents, for whom independent device ownership, peer interaction, school schedules, and exposure to social media platforms increase rapidly. Preschool children aged 2 to 5 years also experience screen exposure, but their media use is more dependent on caregiver mediation and requires a different prevention and regulatory framework.

**Figure 1.**
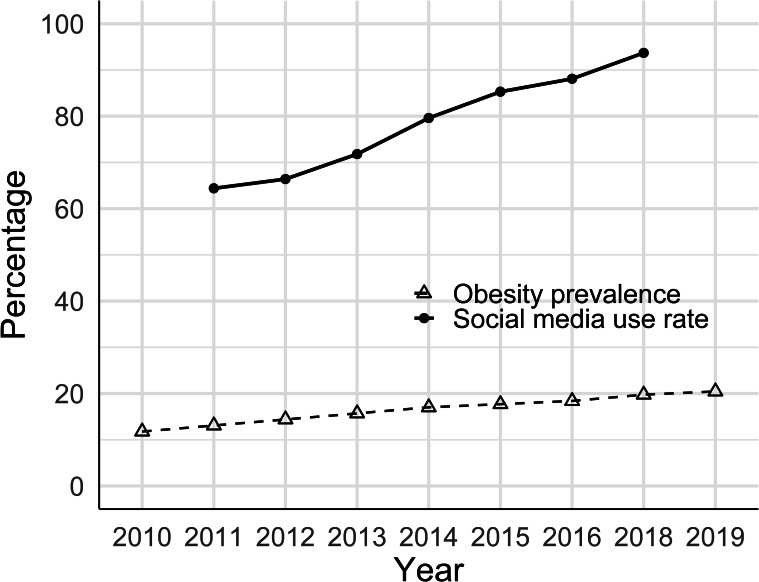
Trends in social media use and childhood obesity in China (2010‐2019). The solid line represents the proportion of children using social media or internet-based platforms, as the China Internet Network Information Center [5] reported. The dashed line indicates the prevalence of childhood obesity, based on data from the Chinese National Survey on Students’ Constitution and Health.

The health risks of obesity are already evident during childhood and adolescence, including metabolic, orthopedic, respiratory, and mental health consequences that may track into adulthood [1,2]. Although lifestyle, environmental, genetic, and socioeconomic determinants remain central, broader technological factors are increasingly relevant to childhood obesity [3]. In China, the prevalence of childhood obesity and youth internet use has increased in parallel over the past decade (Figure 1), suggesting the need to examine social media as an upstream commercial and behavioral environment rather than as a single causal exposure. Traditional prevention strategies focused mainly on individual diet and physical activity have had limited population-level impact [4]. The transition from an individual-centered approach to a more comprehensive, societally driven prevention strategy is urgently needed. This requires rethinking current childhood obesity prevention strategies and exploring the upstream drivers of childhood obesity, such as government regulations regarding the use of social media in China and beyond.

## Key Message 1: Social Media Is a Plausible Obesogenic Environment Among Children

China is home to one of the largest online populations in the world, and children are increasingly engaged with digital platforms [6]. The widespread availability of smartphones, affordable internet access, and the rise of popular social media platforms have drastically changed how children interact with the world. In this Viewpoint, “social media” refers to interactive, internet-based platforms that support user-generated content, social interaction, algorithmic recommendations, targeted content delivery, and live streaming or influencer-based content. Examples in China include WeChat, Weibo, Bilibili, Xiaohongshu, and Douyin (the Chinese version of TikTok). The target audience for this Viewpoint is international, but China is used as a case study because it combines a large youth internet population, rapid platform innovation, and recent regulatory experimentation.

According to the China Internet Network Information Center, 16.7% of China’s 1.108 billion internet users were aged 6 to 19 years in 2024, with a significant portion of their online activities centered on social media platforms [6]. Chinese school-aged children spent an average of 2.77 hours per day on digital screens [7], exceeding the 2-hour recommendation in a Chinese expert consensus statement [8].

The pathways linking social media use to obesity risk are biologically and behaviorally plausible. Screen time can displace physical activity and increase sedentary behavior; passive viewing and scrolling appear more obesogenic than interactive or educational digital activities [9-11]. Digital food marketing can influence preferences, requests, purchases, and consumption of high-fat, high-sugar, or high-salt products, especially when advertising is embedded in influencer content, games, live streaming, short videos, or other digital environments [12-18]. Other pathways include emotional eating, sleep disruption, cyberbullying-related distress, body image pressure, and reward-based platform design. Social media exposure has been associated with unhealthy dietary behaviors, including skipping breakfast, higher intake of snacks and sugar-sweetened beverages, and lower intake of fruits and vegetables [13]. Late-night use may disrupt sleep through behavioral displacement, arousal, and light exposure, while sleep restriction may alter appetite regulation and food reward processing [19,20]. Cyberbullying and appearance-based comparisons can contribute to distress, poor body image, and maladaptive eating behaviors [21]. Reward-based platform design can further encourage prolonged use through continuous novelty, social feedback, and variable reinforcement [22].

The evidence base also has important limitations. Many studies are cross-sectional or observational, and residual confounding is likely because social media use is correlated with socioeconomic conditions, parenting practices, sleep patterns, physical activity, food availability, mental health, and pre-existing obesity. Randomized and quasi-experimental studies that directly test screen time reduction or media-related obesity outcomes are fewer and show mixed results: some interventions reduce screen time, but effects on BMI are generally small or inconsistent [23,24]. Regulations should therefore not be framed as a single-cause solution to obesity. Rather, they represent a precautionary and proportionate response to a commercial and technological environment that can intensify multiple known obesity-related risks.

## Key Message 2: Lessons From Existing Regulation Support Action, but Social Media Requires a Different Policy Design

Public health regulation has previously been applied to upstream determinants of childhood obesity, including food advertising, sugar-sweetened beverages, and breast milk substitute marketing. Restrictions on unhealthy food marketing to children can reduce exposure and are supported by systematic reviews, evidence from television advertising settings, and World Health Organization (WHO) guidance, although implementation and enforcement vary across jurisdictions [15,25-27]. Taxes on sugar-sweetened beverages have reduced purchases in several settings, although translating purchase changes into long-term obesity outcomes depends on the broader dietary substitution and socioeconomic context [28].

**Figure 2.**
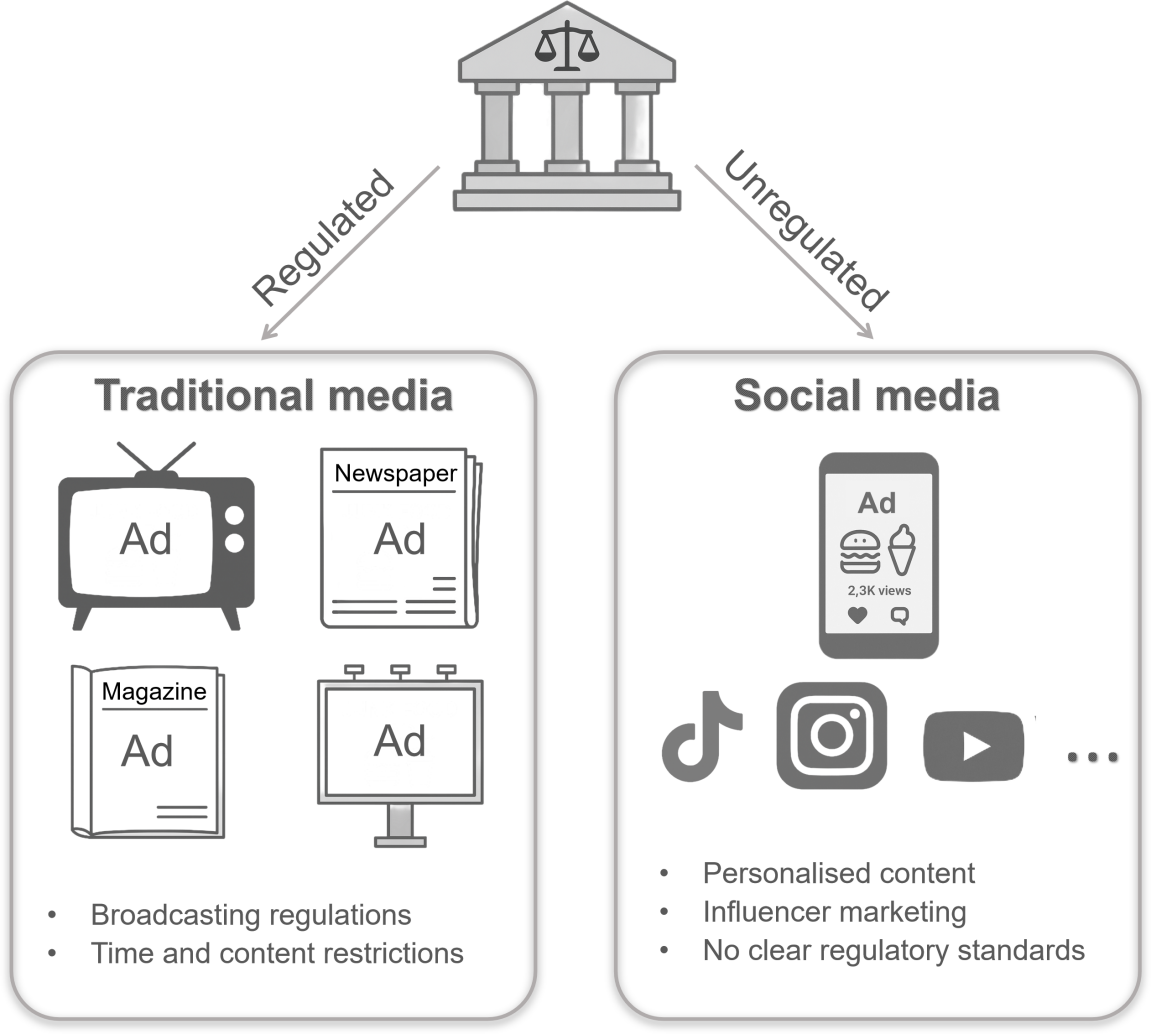
Comparison of regulatory monitoring between traditional media and social media advertising (Ad) targeting children.

Infant feeding policy provides another regulatory lesson. Breastfeeding has been linked to a lower risk of childhood obesity due to its role in regulating children’s metabolic balance [29,30], but evidence linking specific human milk composition to later obesity or body composition is inconsistent [31]. Digital marketing of infant formula and other breast milk substitutes is also a regulatory concern [32]. In China, the government has introduced stricter regulations on the marketing of formula milk to ensure that aggressive marketing tactics do not mislead parents and caregivers, including restrictions on advertising formula milk and a ban on formula companies promoting their products to health care professionals. Research shows that such regulations can help increase breastfeeding rates, which, in turn, can reduce the prevalence of obesity in later childhood [30]. However, human milk can also contain environmental contaminants, and infant feeding policy must consider infection control, maternal health, feasibility, and informed choice rather than present human milk benefits as unconditional or uniform [32]. The relevance to social media regulation is that public health messages should be evidence-based, transparent about uncertainty, and attentive to unintended consequences. Social media differs from traditional media because advertising can be personalized, algorithmically amplified, and embedded in influencer promotions, sponsored content, advergames, livestreams, and brand challenges [14]. These regulatory discrepancies are further illustrated in Figure 2. These features mean that policy cannot simply copy television advertising rules; it must address age assurance, influencer disclosure, algorithmic amplification, platform accountability, data access for independent evaluation, and appeal mechanisms.

## Key Message 3: Regulatory Action Should Be Age- and Context-Sensitive

Regulatory design should distinguish both between minors and adults and within minors by developmental stage. Existing reviews and guidance suggest that age-specific screen time evidence and recommendations differ across early childhood and school age [7,8]. We suggest age-sensitive protections for children aged 6 to 11 years, early adolescents aged 12 to 15 years, and older adolescents aged 16 to 18 years. Younger children require stronger default protections because they have limited advertising literacy and lower autonomy. Early adolescents require protection from persuasive design and influencer marketing while building media literacy. Older adolescents require safeguards that respect growing autonomy, civic participation, education, and social connection. Age segmentation should apply to advertising eligibility, default settings, data collection, persuasive design features, and parental- or school-facing tools.

Several jurisdictions have begun to regulate children’s digital environments, but evidence on health effects remains limited. In China, the 2020 revision of the Law on the Protection of Minors strengthened online protections for minors, and major platforms have introduced youth modes, time limits, content filtering, and parental controls [33]. Australia has implemented a social media minimum age obligation requiring age-restricted platforms to take reasonable steps to prevent users younger than 16 years from holding accounts, with no penalties for children or parents [34]. The European Union’s Digital Services Act requires stronger protection of minors and prohibits targeted advertising to children [35], while several US states have introduced measures related to age assurance, parental consent, school cellphone restrictions, and addictive platform design [36]. However, whether these measures have meaningfully reduced children’s screen exposure or obesity-related digital risks remains unclear because effectiveness depends on age identification, parental activation, and sustained use.

Regulations should also consider social contexts. In urban areas, children may be exposed to sophisticated digital marketing for fast food and food delivery promotions, where online advertising coincides with easy access to unhealthy foods. In rural regions, children living apart from their parents or cared for by grandparents may have less direct supervision. Intergenerational patterns of device use can normalize prolonged screen exposure, and “smartphone parenting” may unintentionally reinforce sedentary digital habits [37]. Policy should therefore combine platform-level rules with support for caregivers, schools, and communities rather than assume that parental responsibility alone can manage algorithmic environments.

## Key Message 4: AI-Assisted Moderation May Help Enforcement but Safeguards Are Essential

AI has the potential to revolutionize the way governments regulate social media content, offering solutions for real-time moderation and more targeted interventions. AI algorithms could be trained to identify and flag content that promotes excessive screen time, junk food, or unhealthy body image ideals, and also help block harmful content in real time, offering a more dynamic approach to regulating digital content. The application of AI in content moderation is already being tested on some platforms, such as detecting and removing harmful content, including hate speech and bullying. A similar approach could be adopted to tackle unhealthy content and advertisements targeted at children on social media. However, AI is not a neutral solution. It can misinterpret context, dialect, humor, satire, health education, or culturally specific content; reproduce biases in training data; overremove legitimate content; miss rapidly changing marketing tactics; and create privacy risks when age assurance or behavioral profiling is used [16,38].

Industry incentives must also be considered. Platforms and advertisers may have stronger technical capacity than regulators and may adapt marketing strategies to avoid detection, for example, through coded language, private groups, live streaming, affiliate links, or creator networks. Therefore, regulation should require transparency reports, independent audits, researcher access to aggregated platform data, clear appeal mechanisms, protection of children’s privacy, and penalties for systematic evasion. AI should support, not replace, human oversight and child rights–based governance.

Predictive analytics is a powerful tool that can help governments design effective policies to tackle childhood obesity, providing valuable insights into the long-term impact of various policy interventions by analyzing patterns in children’s digital behaviors, eating habits, physical activity, and health outcomes. These models can also help identify at-risk populations and target interventions more effectively. Such data-driven approaches could enable more proactive and evidence-based policymaking. To illustrate the potential policy relevance of digital media regulation, we developed exploratory scenario-based projections of obesity prevalence among Chinese boys and girls aged 6 to 18 years from 1995 to 2050 (Figure S1 in Multimedia Appendix 1). Historical prevalence values for 1995, 2010, and 2019 were obtained from the Chinese National Survey on Students’ Constitution and Health [39-41]. The uncontrolled scenario extrapolated sex-specific historical trends, whereas the controlled scenario assumed that comprehensive regulation of obesity-promoting digital exposures would take effect from 2026 onward, using 2025 as the preintervention reference year. Details of the model assumptions, parameter derivation, and calculation procedures are provided in the Supplementary Methods section in Multimedia Appendix 1 [39-41]. These projections are intended to illustrate potential divergence between regulatory and nonregulatory futures rather than to provide formal epidemiological forecasts or causal estimates.

## What Regulatory Measures Are Most Defensible Now?

First, unhealthy food and beverage marketing to minors should be restricted across social media, short-video platforms, games, live streaming, and influencer content. Policies should cover paid advertising, sponsorships, affiliate marketing, product placement, advergames, brand-generated challenges, and algorithmically amplified commercial content. Health claims should be evidence-based and should not exaggerate benefits or obscure nutritional risks.

Second, platforms should provide age-appropriate default settings. For younger users, defaults should limit autoplay, infinite scrolling, late-night notifications, targeted advertising, geolocation-based marketing, and algorithmic recommendations that repeatedly promote unhealthy food or appearance-based content. For adolescents, design should support user control, breaks, content labeling, and privacy-preserving parental or educational guidance rather than covert surveillance.

Third, governments should require platform accountability. This includes independent audits of advertising libraries, access for public-interest researchers to aggregated exposure data, regular reporting of youth exposure to restricted marketing, and evaluation of compliance across urban and rural populations. The policy goal should be a measurable reduction in harmful exposure, not simply the existence of written platform rules.

Fourth, regulation should be embedded in a comprehensive prevention strategy. Schools can teach nutrition, advertising literacy, algorithmic literacy, sleep hygiene, and digital well-being. Family-based approaches can support consistent boundaries and adult modeling of device use [42,43]. Health services can screen for obesity-related risks alongside sleep, mental health, and problematic media use. Technology companies can redesign defaults toward child-centered digital environments.

While this Viewpoint article focuses on China, similar dynamics are observed internationally: high levels of social media engagement among youth, pervasive digital food marketing, and gaps in platform-level governance. Although national-level regulatory measures are crucial, a more coordinated global approach to regulating social media for children is necessary to address childhood obesity effectively. International organizations, such as the WHO, play a crucial role in establishing global standards and advocating for governments to adopt evidence-based policies and regulations to combat childhood obesity. The WHO has already provided guidance on regulating the marketing of unhealthy foods and promoting healthy diets for children. Expanding this guidance to include recommendations for social media regulation could create a more cohesive global strategy for addressing childhood obesity.

## Research and Evaluation Agenda

A scientific Viewpoint paper should identify research needed to support policy. Priority areas include longitudinal studies that measure platform-specific exposure, diet, sleep, activity, mental health, and adiposity over time; randomized or quasi-experimental evaluations of platform defaults, advertising restrictions, and school-based digital literacy; natural experiments following policy changes such as gaming restrictions or age-assurance rules; mechanistic studies of intermediate pathways including sleep, endocrine responses, reward processing, stress, microbiome-related dietary effects, and family food environments; and implementation research on compliance, equity, privacy, and unintended consequences. Future policy should be adaptive: if stronger evidence shows that a specific measure is ineffective or harmful, it should be revised.

## Conclusions

Social media should be recognized as a modifiable obesogenic environment, not as the sole cause of childhood obesity. The strongest current justification for regulatory action is not that social media independently causes obesity, but that algorithmic platforms can amplify several established obesity-related risks at scale, including sedentary behavior, unhealthy food marketing, sleep disruption, body dissatisfaction, cyberbullying-related distress, and persuasive design. Proportionate regulation should prioritize harmful commercial exposure and platform design, be differentiated by developmental stage and social context, and be evaluated transparently. Such regulation should complement education, family engagement, healthy technology design, and regulatory strategies that may change to reflect the latest findings. This balanced approach can make childhood obesity prevention more responsive to the digital era while avoiding overstatement of the current evidence.

## Supplementary material

10.2196/95678 Multimedia Appendix 1 Supplementary methods and supplementary figure illustrating scenario-based projections of obesity prevalence among Chinese children and adolescents.

## Abbreviations

WHO: World Health Organization
