# Supplementary material for: Social Media as a Driver of Obesity in Children and Adolescents (Aged 6-18 Years): It Is Time for Regulatory Action
Source: J Med Internet Res. 2026 Aug 10;28:e95678. doi: 10.2196/95678 (PMC13456531; doi:10.2196/95678)
Supplement: Supplementary file 1 [file jmir-v28-e95678-s001.docx]

### Supplementary Methods: Scenario-based projection model

Historical obesity prevalence among Chinese children and adolescents was obtained from the Chinese National Survey on Students’ Constitution and Health for 1995, 2010, and 2019. Observed prevalence values were 2.0%, 10.9%, and 13.0% for boys, and 1.3%, 5.1%, and 7.0% for girls, respectively [1-3].

For the uncontrolled scenario, sex-specific linear trend models were fitted to the observed prevalence values and extrapolated to 2025, 2030, and 2050. The fitted annual increases were 0.472 percentage points per year for boys and 0.239 percentage points per year for girls. This produced uncontrolled-scenario estimates of 16.66%, 19.02%, and 28.46% for boys in 2025, 2030, and 2050, respectively, and 8.53%, 9.73%, and 14.51% for girls.

For the controlled scenario, comprehensive regulation of obesity-promoting digital exposures was assumed to take effect from 2026 onward, with 2025 treated as the pre-intervention reference year. The controlled trajectory was modelled as an attenuation of the post-2025 incremental increase in obesity prevalence relative to the uncontrolled scenario. The attenuation factor was derived from Veerman et al.’s policy-modelling study of eliminating television food advertising exposure among children [4]. We used the midpoint between the base-case and expert-opinion relative reductions, corresponding to an attenuation of approximately 26.4%. Using this approach, the controlled-scenario estimates were 18.40% in 2030 and 25.35% in 2050 for boys, and 9.41% in 2030 and 12.93% in 2050 for girls. Smooth curves in Supplementary Figure S1 were generated using spline interpolation to connect the observed and projected values for visual presentation.

**References**

1. Sun H, Ma Y, Han D, Pan CW, Xu Y. Prevalence and trends in obesity among China's children and adolescents, 1985-2010. PLoS One. 2014;9(8): e105469.

2. Guo Y, Yin X, Wu H, Chai X, Yang X. Trends in overweight and obesity among children and adolescents in China from 1991 to 2015: a meta-analysis. Int J Environ Res Public Health. 2019;16(23):4656.

3. Yuan C, Dong Y, Chen H, Ma L, Jia L, Luo J, et al. Determinants of childhood obesity in China. Lancet Public Health. 2024;9(12):e1105-e1114.

4. Veerman JL, Van Beeck EF, Barendregt JJ, Mackenbach JP. By how much would limiting TV food advertising reduce childhood obesity? Eur J Public Health. 2009;19(4):365-369.

**Supplementary Figure S1.** Scenario-based projections of obesity prevalence among Chinese boys and girls under uncontrolled and controlled digital-media exposure scenarios, 1995–2050.

Panel A shows boys; Panel B shows girls. Red solid lines represent the uncontrolled scenario, in which obesity prevalence was extrapolated from observed Chinese National Survey on Students’ Constitution and Health values for 1995, 2010, and 2019 using sex-specific linear trend models. Blue dashed lines represent the controlled scenario, assuming comprehensive regulation of obesity-promoting digital exposures after 2026. Under the controlled scenario, the post-2025 incremental increase in obesity prevalence was attenuated for eliminating television food advertising exposure among children. Dots indicate observed or projected anchor values; smooth curves were generated using spline interpolation for visual presentation only. The vertical dotted line marks the beginning of the post-2025 controlled-scenario period.
